# Therapeutic Efficacy by Targeting Correction of Notch1-Induced Aberrants in Uveal Tumors

**DOI:** 10.1371/journal.pone.0044301

**Published:** 2012-08-28

**Authors:** Xiaolin Huang, Li Wang, He Zhang, Haibo Wang, Xiaoping Zhao, Guanxiang Qian, Jifan Hu, Shengfang Ge, Xianqun Fan

**Affiliations:** 1 Department of Ophthalmology, Ninth People's Hospital, Shanghai Jiao Tong University School of Medicine, Shanghai, China; 2 Veterans Affairs Palo Alto Health Care System, Stanford University Medical School, Palo Alto, California, United States of America; 3 Department of Biochemistry and Molecular Biology, Shanghai Jiao Tong University School of Medicine, Shanghai, China; Stanford University School of Medicine, United States of America

## Abstract

There is a need for more effective treatments for uveal melanoma. The recombinant oncolytic adenovirus H101 replicates specifically in p53-depleted tumor cells, and has been approved for use by the Chinese State Food and Drug Administration. However, this treatment is associated with subsequent remission. Transfection of uveal melanoma cells with a small interfering RNA against Notch1 (siNotch1) effectively suppressed Notch1 expression, resulting in significant cell growth inhibition when combined with H101 treatment. Combined treatment with siNotch1 and H101 (H101-Notch1-siRNA) greatly enhanced apoptosis and cell cycle arrest *in vitro* as compared to treatment with H101 or siNotch1 alone. For *in vivo* treatments, the combined treatment of siNotch1 and H101 showed remarkable tumor growth inhibition and prolonged mouse survival in the OCM1 xenograft model. We predict that Notch pathway deregulation could be a feature of uveal melanoma, and could be a therapeutic target, especially if p53 is concurrently targeted.

## Introduction

Uveal melanoma (UM) is the most common primary intraocular malignant tumor in adults, with an incidence of seven cases per million [1], [2]. Despite successful treatment of the primary tumor, nearly 40% of patients die of metastatic disease [3], [4]. UM metastasizes haematogenously and predominantly to the liver. Once metastases are diagnosed, the prognosis is poor, with survival averaging five to eight months [5]. These poor outcomes underline the need for alternatives to traditional treatments such as surgery, radiotherapy and chemotherapy [6], [7].

Recombinant oncolytic adenovirus therapy is an emerging tumor gene therapy [8]. These viruses specifically kill tumor cells while sparing normal cells; recombinant oncolytic adenovirus type 5 (H101) selectively proliferates in TP53 (p53)-deficient tumor cells and specifically lyses tumor cells [9], [10]. This virus-based therapy takes advantage of the fact that the replication and production of adenoviral progeny requires the cell cycle gatekeeper p53 to be inactive, a very frequent characteristic of cancer cells [9]. Both E1B and portions of the E3 region are deleted in this virus. Deletion of a 78.3- to 85.8-μm gene segment in the E3 region, which includes the adenovirus death protein, potentially enhances the safety of the product [11]. The lack of E1B allows H101 to selectively infect and kill tumor cells through specific cell lysis if p53 is mutated [12], whereas H101 does not exhibit a significant cytopathic effect on normal cells in which p53 is active. H101 is the first therapeutic anticancer drug approved for clinical use by State FDA (China) that selectively attacks tumor cells with a modified virus and does not harm healthy cells.

The Notch pathway has been implicated in the generation and development of various tumors [13]. However, the biological mechanism remains unclear. It is well established that the Notch gene encodes a transmembrane heterodimeric receptor [14]. The triggered receptor leads to a series of intracellular molecular signal changes, and the γ-secretase compound which uses Presenilin-1 as a core is a key enzyme in the overall signal pathway. Upon combining with the ligand, the receptor catalyzes the Notch intracellular domain to release, shed, and enter the nucleus. At present, Notch is considered to play an important role in regulating cell growth, cell differentiation, and cell apoptosis [15], [16]. Notch1 expression and activation have been found to be negatively regulated by p53 in several thymoma cell lines [17]. p53 is specifically involved in the control of the Notch1 gene with little or no effect on other Notch gene family members [18], [19], [20]. Importantly, it has been recently reported that Notch signaling promotes growth and invasion in UM [21].

Previously, we demonstrated that blocking Notch1 signaling via RNA interference inhibited HeLa cell growth [22]. It has been reported that targeted knockdown of Notch1 gene expression by a small interfering RNA inhibits the invasion of tumor growth and enhances apoptosis in a variety of tumor cells [23], [24]. We previously used a “double target” approach to antitumor therapy by combining H101 with siRNA that targeted Bcl2 [9], [25]. In this study, we explored the potential synergy of inhibiting Notch signaling combined with H101 oncolytic adenovirus therapy on UM cell lines OCM1 and VUP *in vitro* and *in vivo*. This is the first report of this combination treatment for UM cell lines.

## Results

### Notch1 and p53 status of UM cells

**Figure 1:**
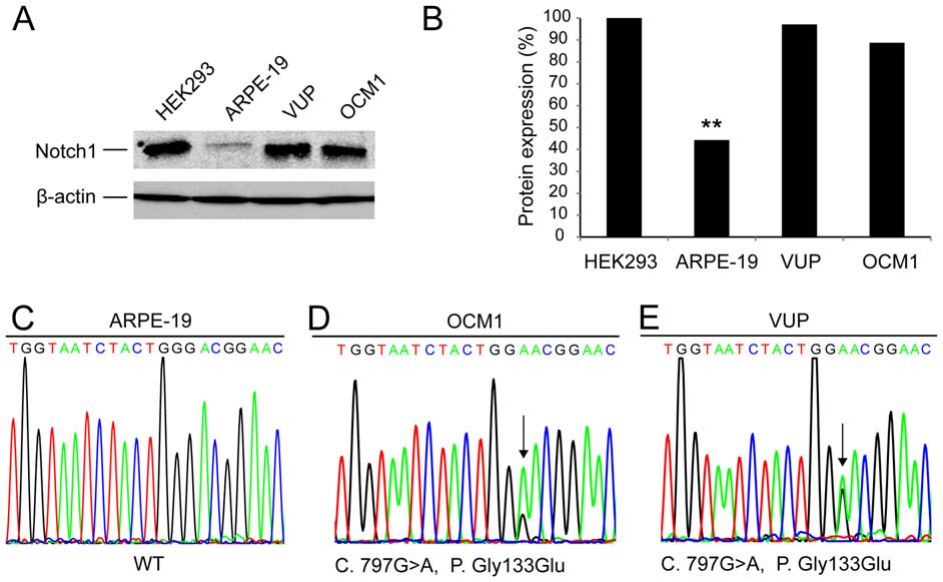
Notch1 and p53 status in UM cells. (A) Western blot analysis of Notch1 expression in UM cells. HEK293, ARPE-19, VUP and OCM1 cells were assessed for Notch1 protein levels. The HEK293 cells were used as positive controls. The normal cell lines ARPE-19 were used as non-malignant controls. Notch1 protein: 120 kDa, β-actin protein: 42 kDa (B) Protein expression were normalized using the internal control β-actin and the positive control band value was set as 1(100%) according to HEK293 cell lines. (C) Sequence analysis of ARPE-19 cell line showing the sequence of wild-type p53 exon 7. (D) and (E) A heterozygous missense mutation of p53 (C. 797G>A, P. Gly133Glu, arrows indicated) was identified in OCM1 and VUP cell lines. Data represent three independent experiments. (*: p<0.05, **: p<0.01).

Notch1 was examined by Western blot in two UM cell lines OCM1 and VUP with human embryonic kidney cells (HEK293) cells as positive controls and human retinal pigmented epithelium cells (ARPE-19) as non-malignant controls. Notch1 was highly expressed in the UM cell lines compared to the ARPE-19 cells (**: *p<*0.01) (Figure 1A, B).

The clinical efficacy of H101 is affected by p53 status. p53 exons 5–8 were sequenced in OCM1 and VUP. Both cell lines contained the same mutation (C. 797G>A, P. Gly133Glu) in exon 7 (Figure 1D, E), while ARPE-19 cell line showed the wild-type sequence of p53 (Figure 1C).

### Sensitivity of UM cells to H101 oncolytic adenovirus

**Figure 2:**
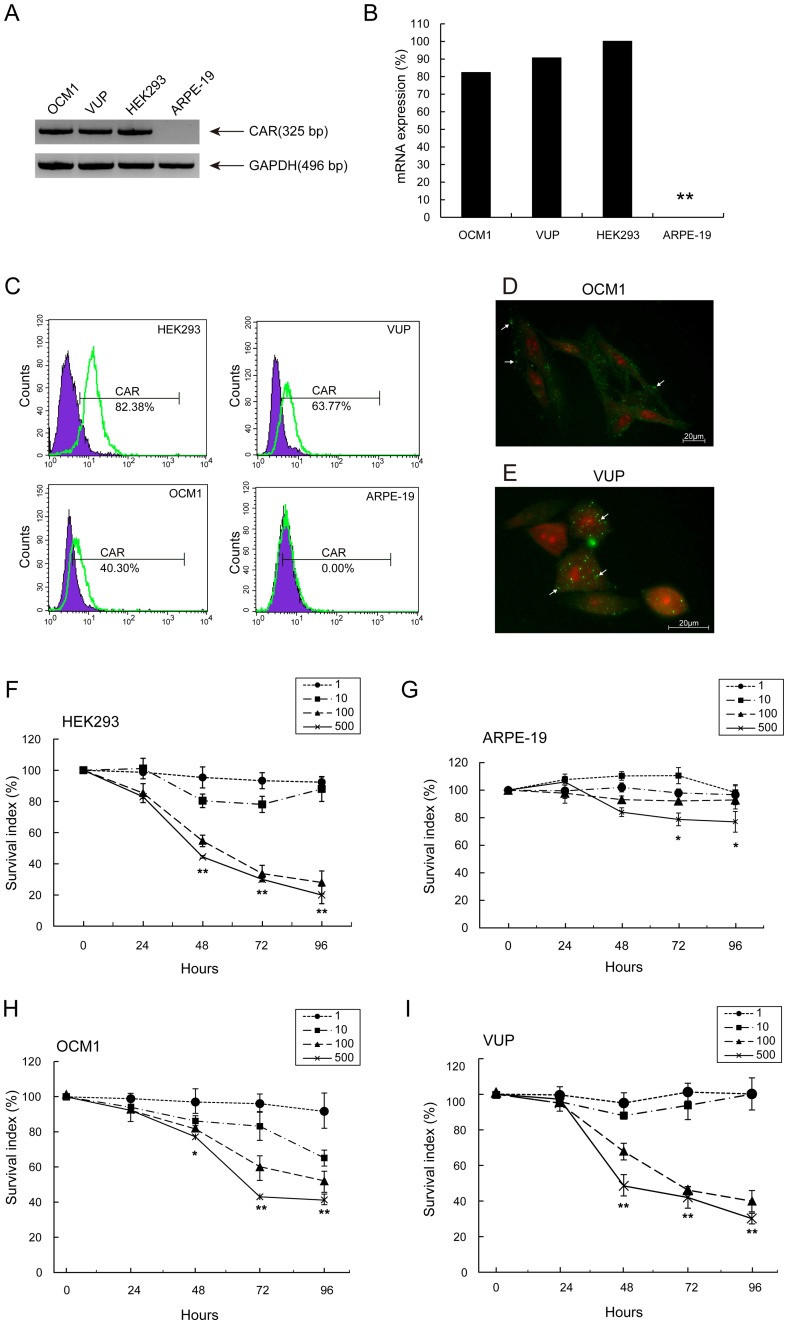
Sensitivity of UM cells to H101 oncolytic adenovirus. (A) RT-PCR analysis of CAR gene in UM cells. The HEK293 cells were used as positive controls. The ARPE-19 cells were used as non-malignant controls. CAR gene: 325 bp, GAPDH gene: 496 bp (B) Densitometric measurement for mRNA expression. The HEK293 band value was set as 100% normalized with the internal control GAPDH. (C) FACS analysis for cell membrane protein CAR. (D) and (E) Immunoﬂuorescence detection of CAR in OCM1 and VUP cells. Nuclei were stained with PI (red), and CAR was visualized with IgG goat anti-mouse secondary antibody (green; white arrows). Infectivity of (F) HEK293, (G) ARPE-19, (H) OCM1 and (I) VUP cells with H101. Cells were incubated in non-FBS culture media and infected with H101 at an MOI of 1, 10, 100, and 500 pfu/cell. The MTT assay was performed at 24, 48, 72 and 96 hours following H101 infection. All data are presented as mean ± SD. of three independent experiments. (*: p<0.05, **: p<0.01, compared with untreated tumor cells).

Coxsackie adenovirus receptor (CAR) has a crucial role in adenoviral infection and is closely related to virus infection rate and efficacy. CAR gene and protein expression in OCM1 and VUP cells were examined by RT-PCR, flow cytometry and immunofluorescence microscopy. CAR was expressed in both cell lines, with higher levels in VUP compared to OCM1 (Figures 2A, B, C, D, E).

We then determined the time- and dosage-dependent killing ability of H101 in HEK293, ARPE-19, OCM1 and VUP cell lines using the MTT assay (Figure 2F, G, H and I). Cells were treated with H101 at various multiplicities of infection (MOI); namely, 1, 10, 100 and 500 [25]. As shown in Figure 2F, H and I, H101 caused significant growth suppression of HEK293, OCM1 and VUP cell lines at MOI of 100 and 500. However, proliferation was not affected at MOI of 1 and 10. The survival index of the non-malignant cell line ARPE-19 was largely unaffected by H101, with only moderate growth suppression observed at an MOI of 500 (Figure 2G). Direct toxic effects may thus be present in all cell lines at higher MOI. In order to minimize such general toxicity, an MOI of 100 was selected for use in subsequent experiments.

### Targeted Notch1 knockdown by synthetic siNotch1

**Figure 3:**
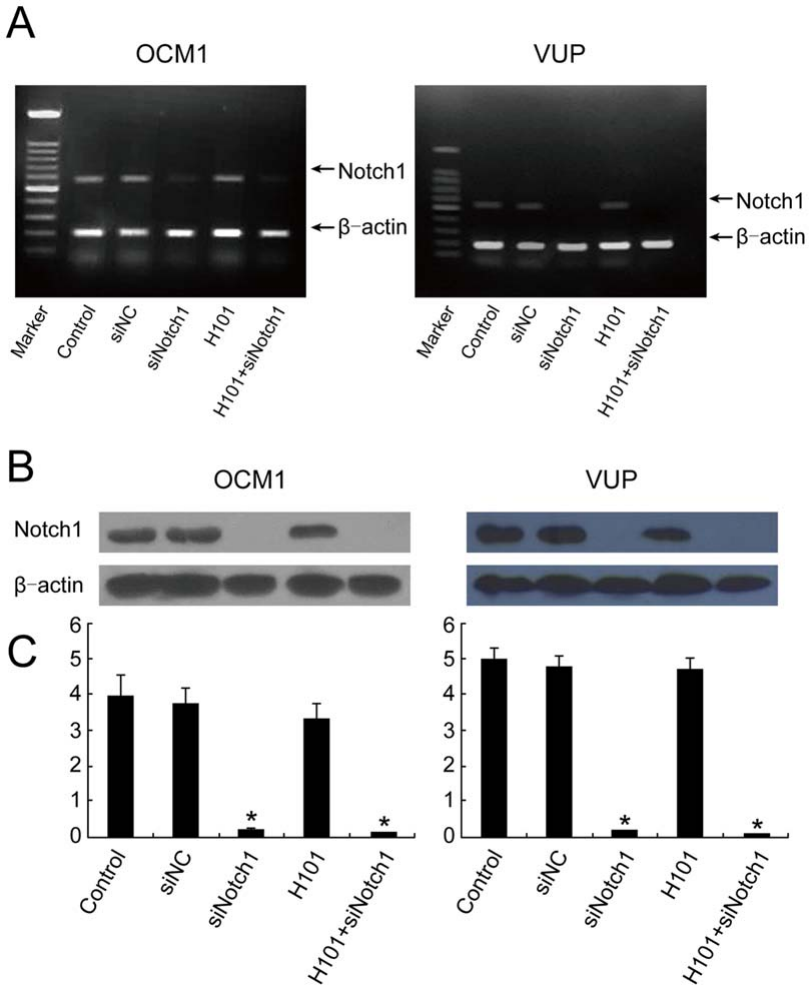
Notch1 gene knockdown by siRNA. (A) RT-PCR results of Notch1 in UM cells. OCM1 and VUP cells were analyzed using specific primers for Notch1 mRNA. A 100 bp DNA ladder molecular marker served as the reference. PCR bands were normalized using the internal control β-actin. (B) Western blot analysis of Notch1 protein in UM cells. All experiments were performed 72 hours following siNotch1(50nmol/L) and control siRNA(50nmol/L) transfection with or without H101 infection (MOI = 100). (C) Western bandScan was used to analyze the gray scale values for different electrophoretic bands, and the relative ratio of the gray scale values between the target Notch1 band and the β-actin internal reference was determined. Notch1 protein: 120 kDa, β-actin protein: 42 kDa.

OCM1 and VUP cells were transfected with siNotch1 [26] and control siRNA (siNC). RT-PCR and Western blot for Notch1 confirmed suppression by siNotch1 but not siNC or by H101 alone. Notch1 expression was markedly inhibited by siNotch1 or siNotch1 plus H101 (Figure 3A, B). The suppression of Notch1 was significant by densitometric measurement (Figure 3C). This experiment confirmed that siNotch1 effectively interferes with Notch1 expression, whereas expression is unaffected by viral infection alone.

### Synergistic suppression of UM cell proliferation by combined H101-Notch1-siRNA

**Figure 4:**
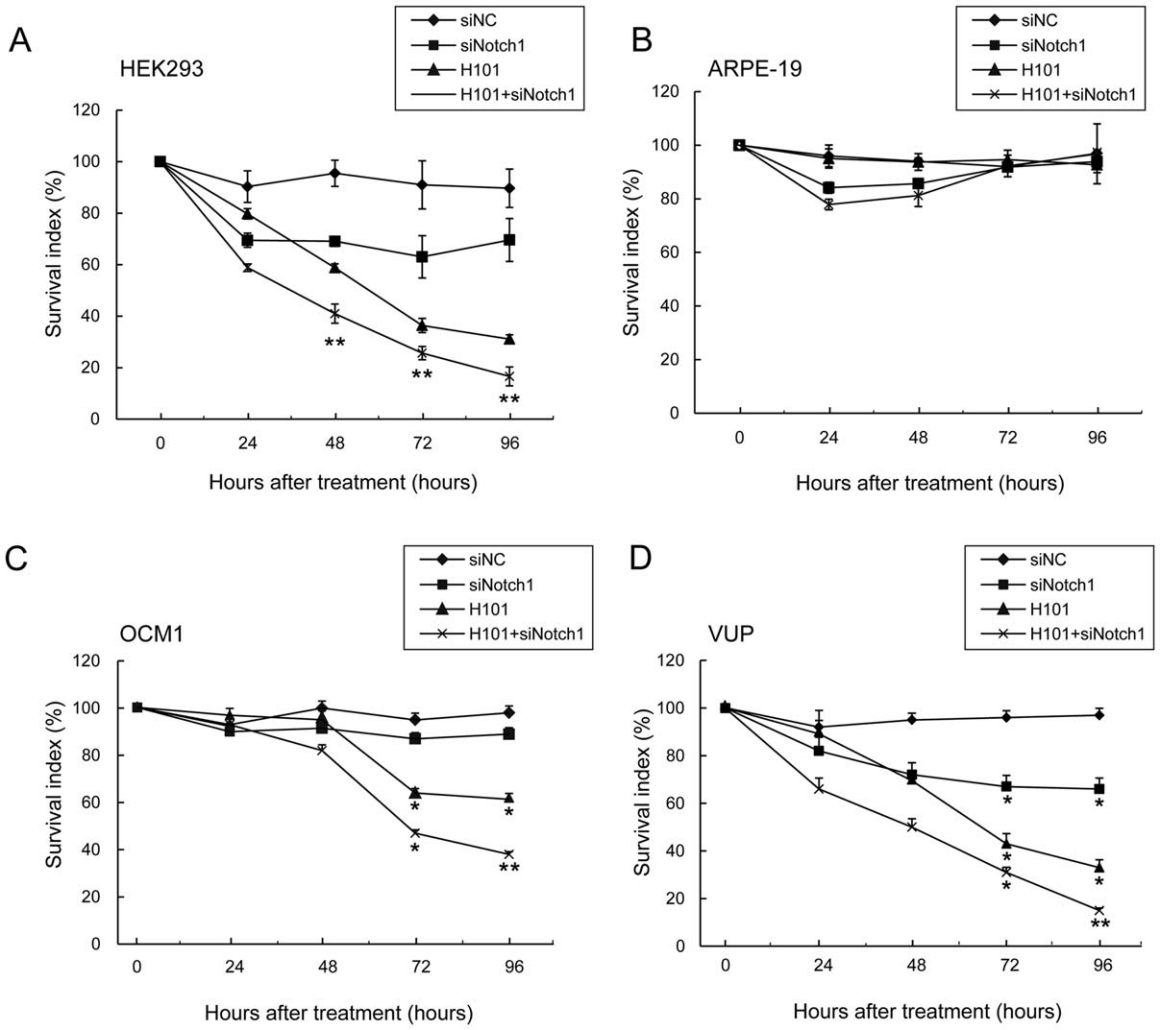
Growth inhibition of combined H101-Notch1-siRNA on UM cells. Survival index of (A) HEK293, (B) ARPE-19, (C) OCM1 and (D) VUP cells by the combined treatment of H101 and siNotch1. Cell survival index was measured by the MTT assay at 24, 48, 72 and 96 hours. Untreated tumor cells were used as controls. SiNotch1 or siNC was used at a concentration of 50nmol/L. H101 infection was performed at an MOI of 100. All data are presented as mean ± SD. of three independent experiments. (*: p<0.05, **: p<0.01, compared with untreated tumor cells).

As seen in Figure 4A, 4C and 4D, monotherapy with siNotch1 or H101 inhibited cell proliferation by a small amount when compared to controls. However, combination H101-Notch1-siRNA treatment produced substantial growth inhibition of HEK293, OCM1 and VUP cell lines (siNotch1/H101/ H101+siNotch1 vs untreated tumor cells, *: *p<*0.05, **: *p<*0.01). However, the combined approach of H101 with siNotch1 as well as monotherapy with either agent alone showed very limited effect on cell growth in ARPE-19 cell line (Figure 4B). After 72 hours, the survival index curve of each treatment was nearly 100%, indicating almost complete recovery of ARPE-19 cells.

### Combined H101-Notch1-siRNA increased S-phase accumulation and apoptosis

**Figure 5:**
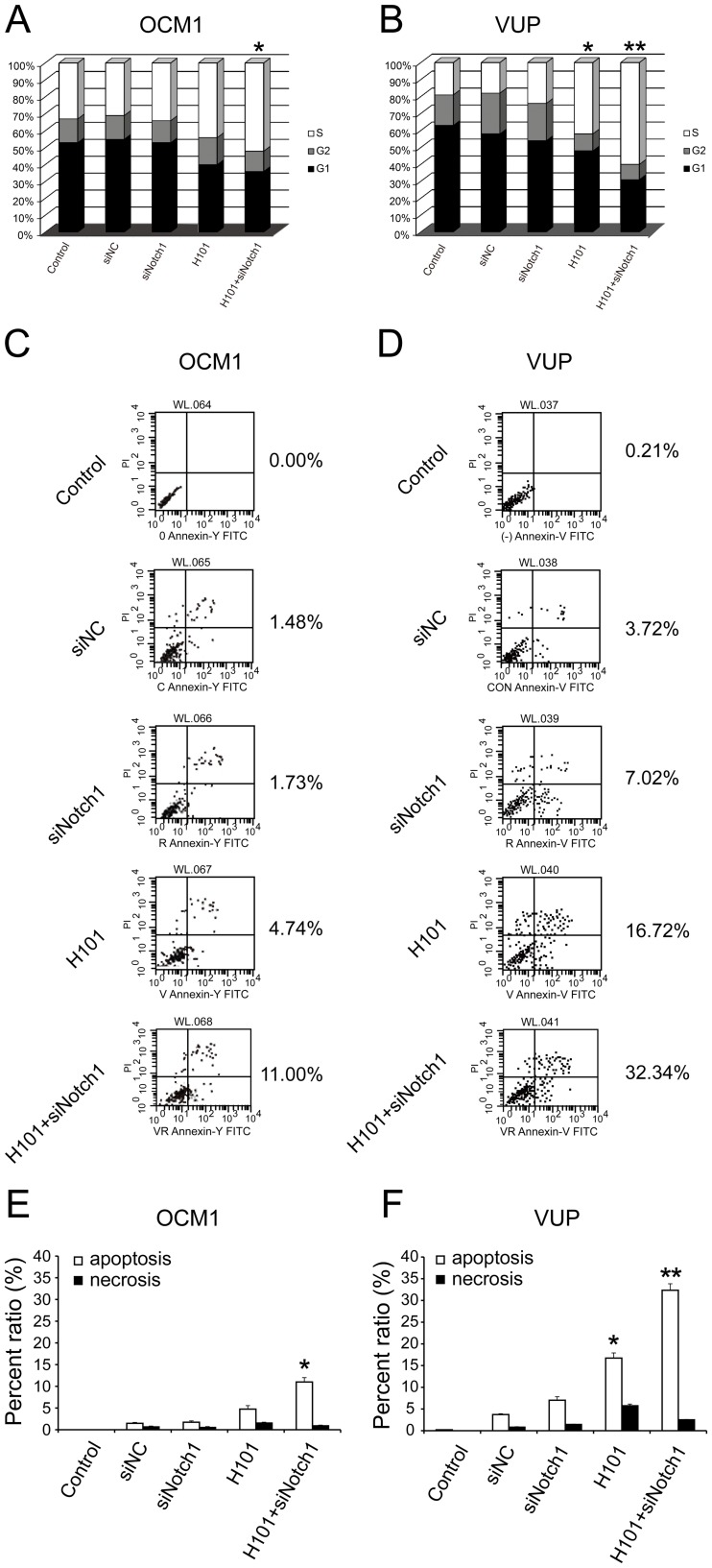
Cell cycle distribution and apoptotic activity of combined H101-Notch1-siRNA on UM cells. (A) and (B) Cell cycle distribution of OCM1 and VUP cells following treatment with siNotch1 and/or H101. OCM1 and VUP cells were harvested 72 hours after co-treatment with siNotch1 (50nmol/L) and H101 (MOI = 100), and propidium iodide staining and FACS analysis were used to analyze the cell cycle distribution. S-phase arrest was detected in the H101 and H101-Notch1-siRNA groups. (C) and (D) Apoptotic activity of OCM1 and VUP cells. Cells were measured by flow cytometry analysis 72 hours after co-treatment with siNotch1 (50nmol/L) and/or H101 (MOI = 100). Upper left: cells affected by necrosis only; upper right: cells affected with both apoptosis and necrosis; lower left: normal cells; lower right: cells affected by apoptosis only. Data are expressed as mean ± SD. of three independent experiments. (*: p<0.05, **: p<0.01, compared with untreated tumor cells). (E) and (F) Relative ratio percentage of apoptosis (cells in lower right group) and necrosis (cells in upper left group) in OCM1 and VUP cells. The percentages of apoptosis and necrosis cells were analyzed according to (C) and (D).

To better understand the mechanism of growth inhibition, the cell cycle status of OCM1 and VUP cells were determined (Figure 5A, B). Cells infected with H101 exhibited moderate accumulation in S-phase. This S-phase accumulation was even more prominent in cells treated with combined H101 and siNotch1, with a corresponding decrease in cells in G1 (*: p<0.05, **: p<0.01). This result is in accordance with the fact that S-phase is the DNA synthesis stage of cells, and there was a synergistic effect on viral replication of combined H101-Notch1-siRNA.

Apoptosis was measured by Annexin-staining and flow cytometry. Monotherapy with siNC, siNotch1, and H101 in OCM1 cells induced apoptosis at 72 hours in 1.48%, 1.73% and 4.74% of cells respectively. While, combined H101 and siNotch1 induced apoptosis in 11% of cells (Figure 5C). VUP cells were more sensitive to apoptosis (Figure 5D); combined treatment induced apoptosis in 32.34% of cells, siNotch1 alone in 7.02%, siNC in 3.72% and H101 alone in 16.72%. These data suggest that the apoptosis level of the H101-Notch1-siRNA combined group was the most significantly augmented (Figure 5E, F).

### *In vivo* antitumor effect by the combined treatment with siNotch1 and H101

**Figure 6:**
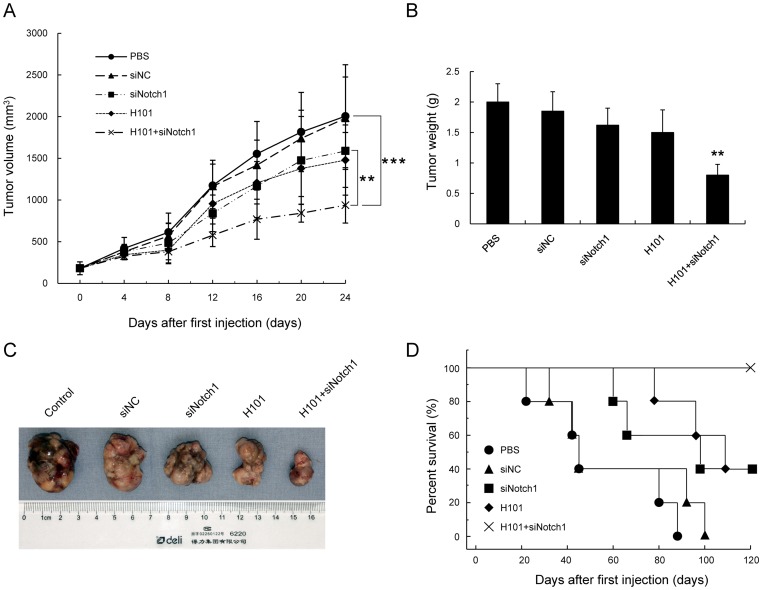
Antitumor effect of combined H101-Notch1-siRNA treatment in an OCM1 tumor xenograft mouse model. (A) Tumor volume following treatments. Subcutaneous tumors were established by implanting OCM1 cells in nude mice (n = 10). (B) Tumor weight on day 24 after first injection (n = 5). (C) Representative pictures of tumor specimens of each treatment group 24 days after first injection. (D) Percentage of mouse survival over 120 days (n = 5). Percent survival was analyzed by Kaplan-Meier survival analysis. Data represent mean ± SD. (**: p<0.01, ***: p<0.001, compared with control group).

In order to apply the *in vitro* findings to the *in vivo* situation, OCM1 cells were implanted into nude mice (n = 10, five groups) (Figure 6). When the volume of the xenografts reached 100–150 mm^3^, we performed intratumor injection of H101 and siNotch1 alone or together (Figure 6A). Treatment with siNC did not result in any suppressive effect on tumor growth. Monotreatment with either siNotch1 or H101 resulted in a moderate inhibition of tumor growth. However, tumor growth was remarkably suppressed in those mice treated with H101 and siNotch1. In addition, on day 24 after first injection, five mice of each group were sacrificed and the tumors were weighed (Figure 6B, C). Monotreatment with siNotch1 or H101 resulted in 19% and 25% reduction in tumor weight respectively. However, the combined treatment of H101 and siNotch1 led to a tumor weight reduction of 61% compared to the PBS group (n = 5, **: p<0.01) (Figure 6B). Representative photographs of tumor specimens of each group were collected. As seen in Figure 6C, the tumor diameter of the combined treatment group was significantly reduced compared with the tumor in the control group.

In order to investigate the long-term therapeutic effects, survival of the remaining mice was examined over a period of 120 days (n = 5; Figure 6D). All mice in the PBS control group and siNC group died by day 88 and day 100, respectively. At the end of the study period, only 40% of the mice treated with H101 or siNotch1 alone were still alive. In contrast, all mice treated with both H101 and siNotch1 survived, and no metastasis was observed (Figure 6D). These results indicate that the combined treatment also resulted in a synergistic antitumor effect *in vivo*.

## Discussion

Traditional approaches to treating UM consist of surgery, radiotherapy and chemotherapy [5], [6]. However, these modes of treatment all have limitations, and are not particularly effective. As such, we set out to explore alternative therapies. The oncolytic adenovirus H101 only replicates in tumor cells in which p53 has been inactivated [9], [10], [12], and thus presents an exciting new cancer therapy modality [7], [9], [10], [11], [27], [28]. We discovered that p53 was mutated in our UM cells, at a site that is also mutated in cutaneous melanoma [29]. This identified UM as a possible therapeutic target for H101. Enhanced cytotoxic effects were observed for the UM cell lines OCM1 and VUP treated with H101 (Figure 4C, D). However, the cell line ARPE-19 was not affected by H101 infection (Figure 4B), as it contains wild-type p53 (Figure 1C). CAR has been identified as a cellular receptor for adenovirus group C serotypes 2 and 5 (AdV2, AdV5) fibers, and for Coxsackie B virus; CAR augments attachment and adhesion of the adenovirus to the cells, and increases susceptibility to virus-mediated gene transfer [10], [30], [31]. We observed high expression of CAR in the UM cell lines OCM1 and VUP, and the higher expression of CAR in VUP cells was consistent with its greater susceptibility to growth suppression by H101 (Figure 2).

Oncolytic adenovirus replication is related to the cell cycle, as replication occurs preferentially during S-phase. As such, viruses often compel the host cell to enter into S-phase. This may be achieved through the generation of basal cell regulators, such as retinoblastoma protein (pRb) and mitotic arrest deficient-like 2 (Mad2), as well as components that interfere with the main surveillance pathways controlled by p53 and ataxia telangiectasia mutated (ATM) [32]. This may explain the accumulation of UM cells in S-phase upon H101 infection. We also observed an increase in apoptosis in transfected cells, especially when combined with siNotch1. This may be related to enhanced viral replication; it may be worthwhile exploring the interaction between Notch1 signalling and virus infection control in future.

There are reported cases of tumor remission following apparently successful H101 monotherapy [33]. In addition, the principal route of administration is through direct intratumoral injection, which limits the clinical application range of adenovirus. Attempts to enhance the therapeutic effect of adenovirus through the joint application of traditional radiotherapy or chemotherapy have augmented responses, but toxicity remains a major limiting factor [27], [28]. Consequently, we investigated whether it was possible to combine H101 with knockdown of certain proto-oncogenes, which may enhance the efficacy of the treatment. In our previous study, we also observed synergistic effect when we combined H101 and siBCL2 in Bcl2 elevated UM cells by enhancing apoptosis and cell cycle arrest through Bax-p53 induced apoptotic pathway [25]. However, some details of the function of siBCL2 remain unclear. In this study, we found that Notch1 is highly expressed in UM cell lines (Figure 1A), in which it acts as a proto-oncogene. This informed our decision to attempt to downregulate Notch1 using a small interfering RNA *in vitro* and *in vivo*.

The Notch pathway regulates the capability of cells to recognize differentiation signals, and plays an important role in regulating cell growth, cell differentiation, tissue renewal, and intracellular environmental stabilization [34]. The Notch protein is a single transmembrane receptor, transformed into as mature heterodimer through proteolysis. Notch activation has been previously implicated in the growth and invasion of UM [21], and the Notch target Hes1 promotes survival of melanocyte stem cells [35]. Uncontrolled expression of Notch and related genes, including the relevant ligand and downstream genes, have been found in many solid tumors (including cervical cancer [36], [37], head and neck cancer [34], [38], renal cancer [39] and breast cancer [40], [41]). Notch is a potential oncogene [42], and an uncontrolled Notch pathway plays an important role in maintaining the phenotype of tumors [21], [43]. Our finding that knockdown of Notch1 inhibited cancer cell growth are consistent with earlier studies [21], [35]. Combined treatment with H101 and siNotch1 augmented the anti-proliferative effect of H101 on UM cells *in vitro* (Figure 4), confirming the potential efficacy of this strategy. We investigated potential safety issues by testing our combinatorial treatment on ARPE-19, a human retinal pigmented epithelium cell line [6], [25], [44]. As shown in Figure 4B, siNotch1 had only a mild effect on ARPE-19 survival, which may be attributed to its low expression of Notch1 (Figure 1A). Consequently, we can be reasonably confident that the treatment will specifically target UM cells, and not the surrounding healthy tissure.

Based on the *in vitro* results, we investigated the effect of combined H101-siNotch1 treatment on growth of OCM1 cell xenografts. *In vivo* experiments indicated that intratumoral injection of H101 together with siNotch1 inhibited tumor growth and prolonged animal survival in nude mice. As such, we argue that H101-siNotch1 may serve as a potential future therapy for UM.

## Materials and Methods

### Ethics Statement

Animal experiments were performed in accordance with institutional guidelines for animal care by Shanghai Jiao Tong University.

### Cell culture

Human UM cell lines(OCM1 and VUP )were kindly provided by Professor John F. Marshall (Tumor Biology Laboratory, Cancer Research UK Clinical Center, John Vane Science Centre, London, UK) [45]. The OCM1 cell line was established from biopsied specimens of choroidal melanomas of spindle B cell type morphology [46]. The VUP cell line was mainly composed of epithelioid cells [47]. ARPE-19 cell line was generously provided by the Department of Ophthalmology, Ruijin Hospital, Shanghai Jiao Tong University School of Medicine, P.R. China. HEK293 cell line was purchased from the American Type Culture Collection (Manassas, VA, USA). OCM1 and VUP cells were cultured in DMEM (Invitrogen, Carlsbad, CA, USA) and HEK293 and ARPE-19 cells were cultured in DMEM? F12 (Invitrogen). Cells were supplemented with 10% fetal bovine serum (FBS) under 5% CO2 at 37°C.

### DNA extraction and p53 sequencing

DNA was isolated using a DNA extraction kit (TaKaRa, Tokyo, Japan). PCR analysis was carried out by using 1 µl of the DNA extract with primers specific for p53 mutation hotspot exons 5–8 (these primer sequences are available upon request). PCR was performed by using the following cycling programme: 5 min at 94°C (30 s at 94°C, 1 min at 56–62°C, 1 min at 72°C) for 35 cycles followed by 5 min at 72°C. PCR products were purified and subjected to single-strand conformation polymorphism analysis.

### Immunofluorescence and Detection of CAR by FACS Analysis

Cells were collected and blocked with normal goat serum (1∶10 dilution in phosphate-buffered saline (PBS) with 0.5% bovine serum albumin (BSA; Invitrogen) at 37°C for 30 minutes. After being washed with PBS twice, cells were incubated with mouse monoclonal antibodies recognizing CAR (1∶50 dilution in PBS with 0.5% BSA; Santa Cruz Biotechnology, Santa Cruz, CA, USA) at 4°C over night. The second day, cells were incubated with goat anti-mouse IgG secondary antibody (DyLight 488; 1∶200 dilution in PBS with 0.5% BSA; Invitrogen) and propidium iodide (PI; 1∶1000 dilution in PBS; BD Biosciences, SanDiego, CA, USA). Cells were placed on coverslips and photographed with a ﬂuorescence microscope at 490 to 520 nm.

FACS analysis (Becton Dickinson, Franklin Lakes, NJ, USA) was used to detect CAR. Cells were collected and washed in FACS buffer containing 2% BSA. After incubating with primary CAR antibody for 1 hour on ice, cells were exposed to secondary antibody (as above). Then cells were analyzed using a FACScan flow cytometry.

### Notch1- siRNA oligonucleotide and Adenovirus H101

According to Masuda S's [26] sequence reported previously, Notch1-siRNA (Forward: 5′ -AAG GUG UCU UCC AGA UCC UGA dTdT- 3′ Reverse: 5′ -UCA GGA UCU GGA AGA CAC CUU dTdT- 3′) and control siRNA (Forward: 5′ -AAA UGU GUG UAC GUC UCC UCC- 3′ Reverse: 5′ -UCA GGU ACU CAG UCA UCC ACA GG- 3′) were synthesized and purified by Shanghai Genepharma (Genepharma, Shanghai, China). Opti-MEM and Lipofectamine 2000 were purchased from Invitrogen.

Recombinant oncolytic adenovirus H101 was kindly provided by Shanghai Sunway Biotech (Sunwaybio, Shanghai, China).

### *In vitro* gene knockdown by siRNA transfection

Tumor cells were seeded at 30–50% confluence in six-well plates 24 hours before siRNA transfection. The cells were transfected with 50 nmol/L siNotch1 or siNC in Opti-MEM using Lipofectamine 2000 reagent following the manufacturer's protocol (Invitrogen). After 6 hours incubation, cells were infected with H101 at a multiplicity of infection of 100. Control group cells were untreated tumor cells in PBS media [9].

### MTT assay

Cells were seeded at 5,000 cells per well in 96-well plates. 20 µl of 5 mg/ml MTT (Sigma-Aldrich, St. Louis, MO, USA) in PBS was added to each well at the end of the incubation time. After 4 hours, media were discarded, and cells were lysed with 100 µl dimethylsulfoxide. Cells were incubated at 37°C with gentle shaking for a further 30 minutes. The optical density was determined with a microplate reader at 570 nm. Absorbance values in the treated groups were normalized to the values of untreated tumor cells to calculate the percentage of survival [10].
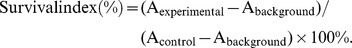



A_experimental_ is the absorbance of the experimental sample, A_control_ is the absorbance of untreated tumor cells sample, and A_background_ is the absorbance of the media. Each experiment was repeated four times.

### Cell Cycle Analysis by FACS

Cells were seeded at 10,000 cells per well in six-well plates. Cells were harvested at 72 hours after H101 (MOI = 100) infection and siNotch1 transfection. Cells were washed twice with PBS and stained with 10 μg/mL PI. Cell cycle distribution was determined by flow cytometry. FACS was performed using a FACScan flow cytometer (Becton Dickinson, Sunnyvale, CA). Data were acquired using CELL Quest software.

### Analysis of Apoptosis

Early apoptosis was detected by staining with Annexin-V-fluorescein isothiocyanate and PI labeling using the Annexin-V-FITC apoptosis kit (BD Biosciences). Tumor cells were analyzed by flow cytometry, as described above.

### Western Blot analysis

Cells were harvested at the indicated time and rinsed twice with PBS. Cell extracts were prepared with lysis buffer, and centrifuged at 15,000 g for 30 min at 4°C. Notch1 proteins were quantified using the BCA Assay Kit (Pierce Biotechnology, Rockford, IL, USA) according to the manufacturer's protocol.

Protein samples were separated by sodium dodecyl sulfate–polyacrylamide gel electrophoresis in 10% (wt/vol) polyacrylamide gels, and transferred to polyvinylidene fluoride membranes (Invitrogen). After blocking with 5% milk for 1 h at room temperature, membranes were incubated with 2 ug/ml antibody in 5% milk overnight at 4°C. The membranes were then incubated with secondary antibody conjugated to a fluorescent tag (Invitrogen). The band signals were visualized and quantified using the Odyssey Infrared Imagining System (LI-COR, Lincoln, NE, USA). The following antibodies were used: Anti-Notch1 monoclonal antibody (Epitomics, CA, USA) and β-actin (Sigma-Aldrich).

### *In vivo* antitumor effect by the combined treatment with H101 and siNotch1

Female athymic 5-week-old nude mice were deeply anesthetized and 1×10^7^ OCM1 cells were subcutaneously injected into their right flank. Tumor-bearing mice were randomly assigned into five groups of 10 mice each. Mice then received intratumoral injections of either PBS (control), 10 µg of siNC or siNotch1, 1×10^8^ plaque forming units of H101, or both H101 and siNotch1. All treatments were performed every three days (on day 1, 4, 7, 10, 13, 16 and 19 after first injection), for a total of seven injections. Tumor growth was monitored using a caliper every four days. Tumor volume was calculated using the formula: 0.54× length (mm) × width (mm) × height (mm).

Five mice of each group were sacrificed, and the tumors were weighed. The remaining mice were observed for 120 days to determine the survival rate.

### Statistical Analysis

SPSS 11.0 statistical software was used to perform statistical analysis. Student's t-test was used to compare two means, and one-way ANOVA was used to compare more than two means. Animal survival after treatment was analyzed by Kaplan-Meier survival analysis. P<0.05 was considered statistically significant. Values were expressed as means ± SD.
